# The paddle effect on speed perception: A multilevel Bayesian meta-analysis

**DOI:** 10.3758/s13423-026-03011-5

**Published:** 2026-09-10

**Authors:** Wenbo Luo, Lunbo Zhang, Yuki Yamada

**Affiliations:** 1 Graduate School of Human-Environment Studies, Kyushu University, 744 Motooka, Nishi-ku, Fukuoka, 819-0395 Japan; 2 Faculty of Arts and Science, Kyushu University, Fukuoka, Japan

**Keywords:** Paddle effect, Speed perception, Action-specific perception, Bayesian meta-analysis, Three-level model

## Abstract

The paddle effect is considered one of the most robust phenomena in action-specific perception. In computer game-based Pong tasks, participants perceive targets as moving more slowly when using a larger paddle compared to a smaller one. We employed multilevel Bayesian modeling to conduct the first meta-analysis of this literature. A systematic search identified 26 independent studies from 13 articles, with a total sample size of *N* = 542. Results provided extreme evidence for the existence of an overall paddle effect, Hedges’ *g* = 0.80, 95% CrI [0.71, 0.88]. Furthermore, Bayesian meta-regression analyses indicated that the effect was not moderated by experimental paradigms or measures, supporting its methodological robustness. The present meta-analysis provides the first quantitative synthesis of evidence for the paddle effect and establishes a foundation for its continued characterization in future research. In accordance with open science practices, the data, R code, and Supplementary Materials are available at the Open Science Framework (https://osf.io/fxvbh/overview?view_only=a636518ac0f747eab10780f0e70f1e2d).

## Introduction

The action-specific perception account challenges the traditional view that perception provides a veridical representation of the environment (e.g., Pylyshyn, 1999), suggesting instead that people perceive the environment in terms of their ability to act (Proffitt, 2006; Witt, 2011a). According to this account, perceptual experiences are modulated by factors related to one’s diminished or augmented physical capabilities. For example, observers overestimate egocentric distances when wearing a heavy backpack (Proffitt et al., 2003), whereas they perceive target objects as closer when using a reach-extending tool (Suh & Abrams, 2018; Witt, 2011b; Witt et al., 2005).

However, some studies failed to replicate the backpack effect (e.g., Hutchison & Loomis, 2006; Woods et al., 2009) and the tool-use effect (e.g., de Grave et al., 2011). This lends support to the claim that such action-specific effects may be due to experimental demand characteristics rather than perceptual distortions (Durgin et al., 2009; Firestone, 2013). Firestone and Scholl (2016) further proposed a framework arguing that action-specific perceptual distortions are not true top-down effects. They identified six potential pitfalls and suggested that many studies fall into at least one of them. These pitfalls primarily involve nonperceptual factors, such as post-perceptual judgment, attentional effects, and particularly response bias.

**Fig. 1:**
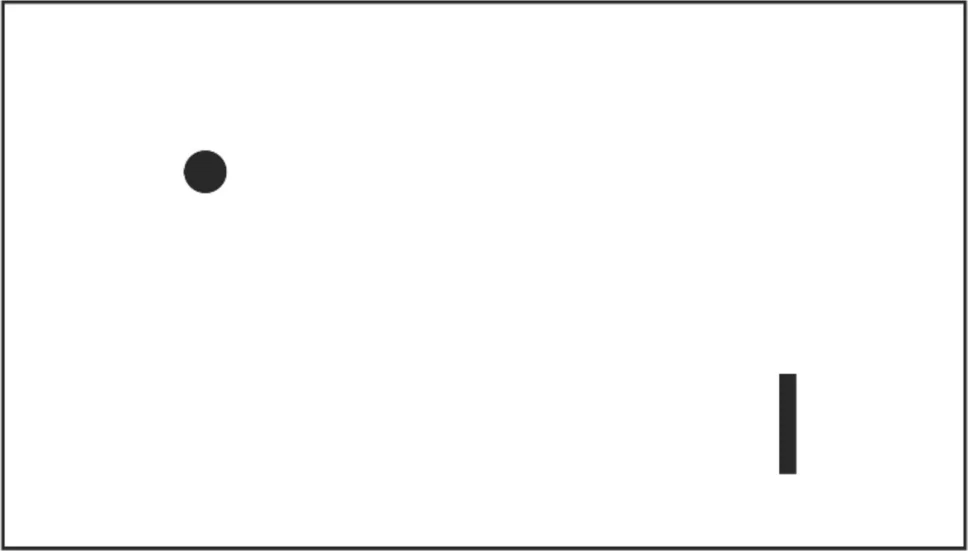
Schematic illustration of the Pong task. In each trial, participants use paddles of varying sizes to block a target ball moving across the screen

Witt and colleagues (2016) pointed out that the paddle effect avoids all six pitfalls. The paddle effect is considered one of the most robust action-specific effects and has been independently and successfully replicated (Kirsch & Kunde, 2018). Participants, in tasks modified from the computer game Pong, use paddles of varying sizes to block a moving target (Fig. 1). The perceived speed of the target differs as a function of paddle size. Specifically, participants perceive the target as moving more slowly when using a larger paddle, which is more effective at blocking, than when using a smaller paddle (Witt & Sugovic, 2010, 2012).

Researchers have employed two main paradigms to test the paddle effect. In the control-based paradigm, participants actively move a paddle vertically to block a moving target (e.g., Witt & Sugovic, 2010, 2012). In contrast, the launch-based paradigm requires participants to judge the launch timing of the paddle to intercept the target (e.g., Witt & Sugovic, 2013a). Both paradigms have reliably demonstrated the paddle effect. Importantly, the paddle effect has been shown to be robust across multiple measures, such as speed bisection tasks (e.g., Witt & Sugovic, 2010), speed ratings tasks (e.g., Witt & Sugovic, 2012), and action-based measures (e.g., Witt & Sugovic, 2013a).

A central controversy regarding the paddle effect concerns its action-specific interpretation. Kirsch and Kunde (2018), in addition to their replication, provided evidence that the paddle effect persists even when participants passively observe a computer-controlled paddle without motor involvement. This raises the question of whether action capabilities alone can account fully for this effect. Yet, empirical findings remain inconsistent; for instance, Witt et al. (2012) found that the paddle effect was not observed under computer-controlled conditions.

While the paddle effect is a representative action-specific phenomenon, there has not yet been a comprehensive meta-analysis focused on this effect. To address this gap, we conducted a meta-analysis integrating the relevant findings. The aims of the present study were threefold. First, we quantified the overall effect size to evaluate the existence and robustness of the effect. Second, we examined whether the degree of motor involvement moderates the paddle effect. In control-based paradigms, participants continuously control the paddle’s position to track and block the target, whereas they only initiate a single launch by pressing a button in launch-based paradigms. Thus, the control-based task demands a higher degree of motor involvement. If the paddle effect does not depend on action-related processes, the effect sizes between the two paradigms should be comparable. Although this analysis cannot directly resolve the theoretical debate over whether the paddle effect stems from one’s ability to act—a question requiring further empirical investigation—it provides indirect evidence for the potential contribution of motor involvement to the effect. Finally, we tested whether the paddle effect is moderated by response bias. If the paddle effect is partly attributable to such bias, studies employing explicit measures (speed bisection tasks) should exhibit larger effect sizes than those using action-based measures.

## Method

### Literature search

This study followed the Preferred Reporting Items for Systematic Reviews and Meta-Analyses (PRISMA) guidelines (Page et al., 2021). We systematically searched for relevant studies in PsycINFO via Ovid, Web of Science, Scopus, PubMed, and ScienceDirect. The search terms included (“pong effect” OR “paddle effect” OR “action-specific effect*” OR “action-specific perception”). Detailed search strategies are reported in the Supplementary Materials. The initial search was conducted on August 21, 2025, and yielded 994 records. After removing duplicates, 522 records were retained. To minimize the risk of missing relevant literature, we further performed a citation search on the studies included in the full-text review, which identified an additional 250 records.

In accordance with best-practice guidelines (Higgins et al., 2024), we also conducted a grey literature search targeting non-peer-reviewed studies, as the inclusion of grey literature can reduce the risk of exaggerated effect size estimates (McAuley et al., 2000). Using similar search terms, we searched ProQuest Dissertations & Theses A&I and Google Scholar. As of August 21, 2025, the former returned 78 records, and we screened the top 100 records from the latter.

### Eligibility criteria

Studies were included only if they met the following criteria:

1. The study must be empirical. Literature reviews, replies, and commentaries were not included.
2. The study must employ a Pong task, with paddle size as an independent variable and perceived speed as a dependent variable. Studies investigating other manipulations (e.g., tool-use) or other perceptual dimensions (e.g., distance or size), within the action-specific perception literature, were excluded.
3. The study must involve direct measurement of participants’ own action capabilities in using a paddle to attempt to block the target. Studies that involved non-self-paddle manipulation in which participants observed another person or a computer performing the Pong task were excluded. This criterion was applied at the study-design level. If a paradigm combined self-controlled conditions with such non-self-control conditions, the study was excluded.
4. The study must not be a duplicate publication. If the same data were reported in multiple publications, only one study was included. In cases where a thesis/dissertation was also published as a journal article with the same data, the journal article was used; otherwise, the thesis/dissertation was used.
5. The study must report sufficient statistics to allow for the calculation of effect sizes.

**Fig. 2:**
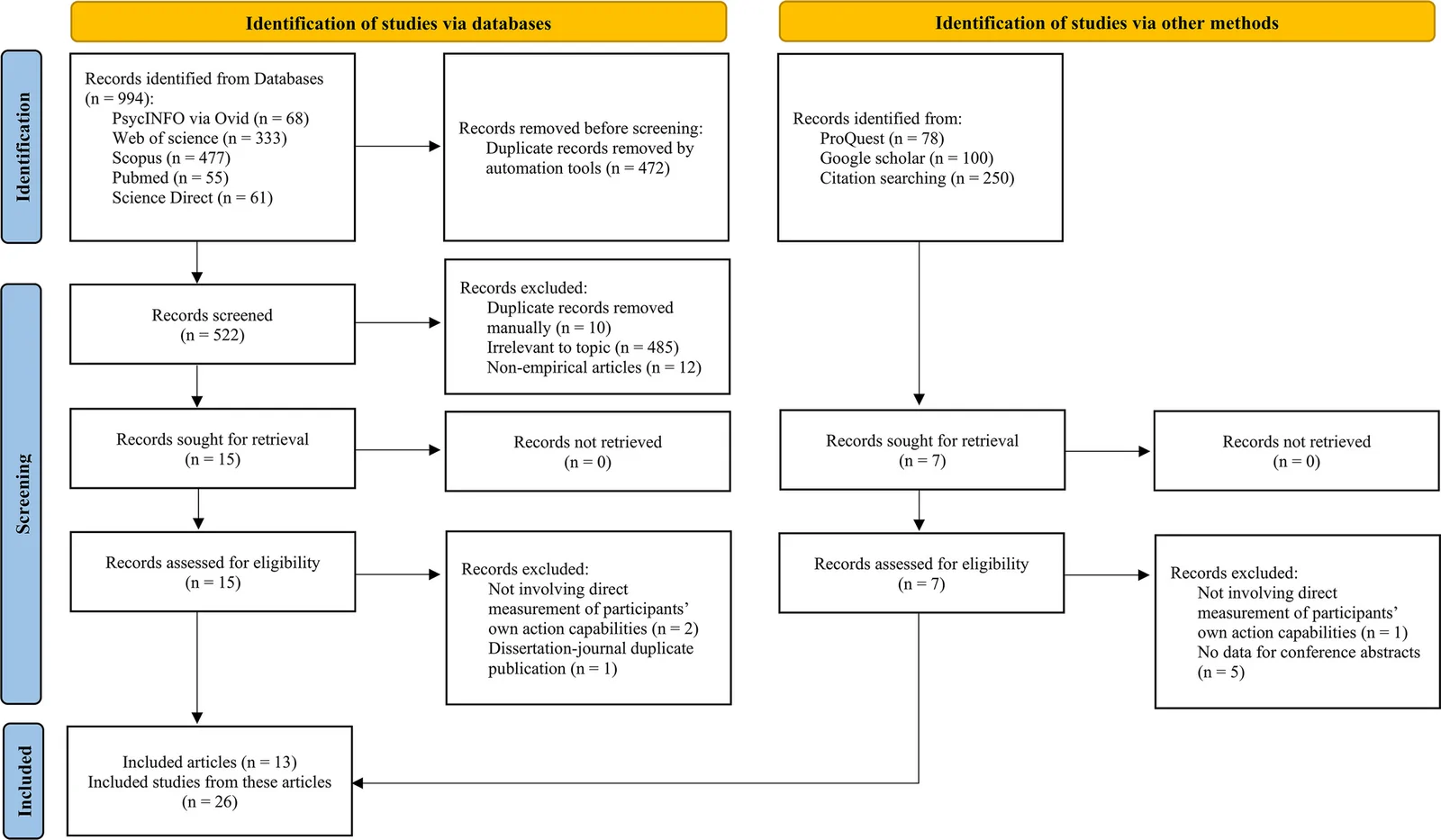
PRISMA flow diagram showing the process of study identification, screening, and inclusion

Based on these inclusion and exclusion criteria, a total of 26 studies from 13 articles (12 journal articles and one thesis) were included. Figure 2 illustrates the comprehensive retrieval procedure. A separate spreadsheet listing all retrieved records, screening decisions, and reasons for exclusion is available on the Open Science Framework.

### Coding of studies

Two independent authors (the first and second authors) initially coded the basic information from two articles. Any discrepancies identified during this pilot stage were resolved through discussion, and, if necessary, by consulting the third author. This process was used to finalize the coding scheme. Subsequently, the same two authors independently coded all studies included in the present meta-analysis. The coded study characteristics (see Table 1) included author and publication year, source type, sample size, paradigm (control-based or launch-based), and measure (e.g., speed bisection task or action-based measure). Intercoder reliability, then, was assessed using the percentage of agreement. Initial agreement reached 100% for all variables, except for measure (93.5%). All discrepancies were subsequently resolved through discussion between the two authors.

**Table 1:** Characteristics of studies included in the present meta-analysis

Author & publication year	Source type	Study no.	*N*	Paradigm	Measure
King (2016)	T	S3	24	Control	Speed bisection task
King et al. (2018)	J	S1	23	Control	Speed bisection task
		S2	14	Control	Speed bisection task
Kirsch & Kunde (2018)	J	S1	12	Launch	Speed comparison task
Laitin & Witt (2020)	J	S1	34	Control	Speed bisection task
Witt (2017)	J	S1	43	Control	Speed bisection task
		S2	36	Control	Speed bisection task
		S3	66	Control	Speed bisection task
Witt (2018)	J	S1(m1)	13	Launch	Speed bisection task
		S1(m2)			Action-based measure
		S2	14	Launch	Action-based measure
Witt & Sugovic (2010)	J	S2	14	Control	Speed bisection task
		S3	12	Control	Speed bisection task
		S4	7	Control	Speed bisection task
Witt & Sugovic (2012)	J	S1	11	Control	Speed rating task
		S2	10	Control	Speed comparison task
		S3	14	Control	Speed rating task
		S4	9	Control	Speed bisection task
		S5	10	Control	Speed bisection task
Witt & Sugovic (2013a)	J	S1(m1)	12	Launch	Speed rating task
		S1(m2)			Action-based measure
		S2(m1)	12	Launch	Speed bisection task
		S2(m2)			Action-based measure
Witt & Sugovic (2013b)	J	S1	35	Control	Speed bisection task
Witt et al. (2016)	J	S1(m1)	24	Launch	Speed bisection task
		S1(m2)			Action-based measure
		S2(m1)	17	Launch	Speed bisection task
		S2(m2)			Action-based measure
Witt et al. (2017)	J	S1	43	Control	Speed bisection task
Witt et al. (2018)	J	S1	16	Control	Speed bisection task
		S2	17	Control	Speed bisection task

Studies included in the present meta-analysis are marked with an asterisk in the reference list. J = journal article; T = thesis. Suffixes (m1, m2) indicate multiple measures within the same study conducted with the same participant sample. All included studies employed a within-subjects design

### Effect-size computation

For all studies, we used Cohen’s *d* as the effect size. To this end, we computed the standardized mean difference in estimated target speed between different paddle-size conditions. A positive *d* indicates that, in the Pong task, participants perceived the target as moving more slowly when using a larger paddle compared to a smaller one. Effect sizes were calculated using the formulas provided by Cooper et al. (2019); these detailed formulas are provided in the Supplementary Materials.

Whenever possible, we prioritized the use of statistical data reported in the original studies to compute effect sizes. For studies in which data had to be extracted from graphical displays, the first and second authors independently used WebPlotDigitizer (Rohatgi, 2024), a tool whose use has demonstrated high inter-rater reliability and validity (Drevon et al., 2017). If discrepancies greater than 10% occurred between authors, the first author re-extracted the data, and the final values were calculated as the average of the two closest extractions. Since Cohen’s *d* tends to be overestimated in small samples, it was converted to Hedges’ *g* by multiplying Cohen’s *d* by the correction factor *J* (Hedges, 1981):

$$J=1-\frac{3}{4df-1}$$

### Data analysis

#### Bayesian meta-analytic model

We employed a three-level meta-analytic model to adequately account for the dependence issue (Cheung, 2014; Van den Noortgate et al., 2013). Several included articles contained more than one study, and moreover, due to experimental manipulations, several studies yielded multiple effect sizes. This structure, where effect sizes are nested within studies and those studies are, in turn, nested within articles, results in dependence among effect sizes originating from the same study or article (Van den Noortgate et al., 2013). In the three-level model, sampling variance was modeled at Level 1, studies at Level 2, and articles at Level 3 (see Supplementary Materials), thus allowing us to model the dependence both between articles and between studies nested within them. To conform to the assumptions of the three-level structure (Van den Noortgate et al., 2013), we performed an aggregation for effect sizes from the same study such that each independent sample corresponded to a single outcome (Cheung, 2014). Specifically, for studies employing three paddle sizes, we calculated a weighted average of the three pairwise effect sizes using the inverse of their respective variances as weights, following the approach used in a recent action-specific meta-analysis (Molto et al., 2020).

The three-level model was estimated under a Bayesian framework. We assigned weakly informative priors for all model parameters. For the overall effect size (α), which represents the model’s grand intercept, we assigned a Normal distribution, α ∼ Normal(0, 1), considering that effect sizes in psychological research typically range from 0 to 1.5 (Szucs & Ioannidis, 2017). For all heterogeneity parameters (the standard deviations of the random effects), we specified HalfCauchy distributions, in particular, τ_article_, τ_study_ ∼ HalfCauchy(0, 0.1). This distribution possesses suitable features for psychological research (Williams et al., 2018) and inherently restricts the standard deviation estimates to be positive. Following Molto et al. (2020), the three-level Bayesian meta-analytic model is formally specified as follows:

$$\begin{array}{c}{y}_{ij} \sim \mathrm{N}\mathrm{o}\mathrm{r}\mathrm{m}\mathrm{a}\mathrm{l}({\upmu}_{ij}, {\upsigma}_{ij})\\ {\upmu}_{ij}=\upalpha +{\upalpha}_{\mathrm{a}\mathrm{r}\mathrm{t}\mathrm{i}\mathrm{c}\mathrm{l}\mathrm{e}[j]}+{\upalpha}_{\mathrm{s}\mathrm{t}\mathrm{u}\mathrm{d}\mathrm{y}[ij]}\\ \begin{array}{c}{\upalpha}_{\mathrm{a}\mathrm{r}\mathrm{t}\mathrm{i}\mathrm{c}\mathrm{l}\mathrm{e}[j]} \sim \mathrm{N}\mathrm{o}\mathrm{r}\mathrm{m}\mathrm{a}\mathrm{l}(0, {\uptau}_{\mathrm{a}\mathrm{r}\mathrm{t}\mathrm{i}\mathrm{c}\mathrm{l}\mathrm{e}})\\ {\upalpha}_{\mathrm{s}\mathrm{t}\mathrm{u}\mathrm{d}\mathrm{y}[ij]} \sim \mathrm{N}\mathrm{o}\mathrm{r}\mathrm{m}\mathrm{a}\mathrm{l}(0, {\uptau}_{\mathrm{s}\mathrm{t}\mathrm{u}\mathrm{d}\mathrm{y}})\\ \begin{array}{c}\upalpha \sim \mathrm{N}\mathrm{o}\mathrm{r}\mathrm{m}\mathrm{a}\mathrm{l}(0, 1)\\ {\uptau}_{\mathrm{a}\mathrm{r}\mathrm{t}\mathrm{i}\mathrm{c}\mathrm{l}\mathrm{e}}, {\uptau}_{\mathrm{s}\mathrm{t}\mathrm{u}\mathrm{d}\mathrm{y}} \sim \mathrm{H}\mathrm{a}\mathrm{l}\mathrm{f}\mathrm{C}\mathrm{a}\mathrm{u}\mathrm{c}\mathrm{h}\mathrm{y}(0, 0.1)\end{array}\end{array}\end{array}$$

### Bayesian statistical analysis

Using this Bayesian modeling approach, we estimated the overall paddle effect (α), the heterogeneity between articles (τ_article_), and the heterogeneity between studies nested within articles (τ_study_). Here, τ represents the standard deviation. All analyses were conducted in R (R Core Team, 2025) using the *brms* package (Bürkner, 2017), which interfaces with Stan (Stan Development Team, 2025).

To further quantify the evidence for competing hypotheses regarding the overall paddle effect, we computed the Bayes factor using brms, which internally relies on the *bridgesampling* package (Gronau et al., 2020). We compared the basic meta-analytic model (including the intercept) with a null model in which the intercept was fixed to zero. Because the Bayes factor is the ratio of the marginal likelihoods of these two models, it can be interpreted as the relative strength of evidence for one model over the other (Kass & Raftery, 1995). In the present study, we reported BF_10_ and interpreted it according to the Bayesian evidence classification guidelines (Lee & Wagenmakers, 2013; for details, see Supplementary Materials).

We also computed the 95% credible intervals (CrI). These intervals represent the range within which the true effect size lies with 95% posterior probability, given the data and the priors (Morey et al., 2016). This interpretation is distinct from that of frequentist confidence intervals (see Nalborczyk et al., 2019).

For each model described previously, we ran four Markov Chain Monte Carlo (MCMC) chains, each consisting of 20,000 iterations, with the first 5,000 iterations discarded as warm-up. We assessed posterior convergence via the trace plots and the potential scale reduction factor $\widehat{\mathrm{R}}$ (Gelman & Rubin, 1992). Convergence is indicated by $\widehat{\mathrm{R}}$ values close to 1.00 (i.e., < 1.01).

#### Sensitivity analysis

We conducted leave-one-out sensitivity analyses at both the article (Level 3) and study (Level 2) levels to evaluate the robustness of the overall paddle effect. First, we removed one article at a time and refitted the basic meta-analytic model. Next, we removed one study at a time and repeated the same procedure. Robustness was assessed by examining whether the estimated overall effect size changed substantially across these iterations.

#### Moderator analysis

We examined the potential influence of two categorical moderators, paradigm and measure, on the overall paddle effect. We fitted two separate Bayesian meta-regression models, one for each moderator. The MCMC sampling parameters and the priors for the intercept and heterogeneity components were identical to those used in the basic meta-analytic model. For the moderator coefficient β, we assigned a weakly informative prior, Normal(0, 0.5). The categorical moderators were processed using contrast coding (−0.5 and 0.5), such that the model’s intercept represented the grand mean effect size. For each moderator, we estimated β and its 95% CrI. The corresponding BF_10_ was computed by comparing the model including the moderator against the basic meta-analytic model. To assess the sensitivity of the results to the prior for β, we repeated both moderator analyses using a narrower and a wider prior, Normal(0, 0.25) and Normal(0, 1), respectively.

#### Publication bias

Publication bias was first assessed using the *metafor* package (Viechtbauer, 2010). We visually inspected the funnel plot (Sterne et al., 2005) and used a contour-enhanced funnel plot (Peters et al., 2008) to help distinguish asymmetry due to publication bias from that due to other sources of heterogeneity. A multilevel Egger’s regression test (Egger et al., 1997; for the multilevel extension, see Fernández-Castilla et al., 2021) was then conducted as a formal statistical check, accounting for the nesting of studies within articles; a *p* value less than .05 suggested potential publication bias.

We subsequently supplemented our analyses with a modern sensitivity analysis approach implemented in the *PublicationBias* package (Braginsky et al., 2023; for a methodological discussion, see also Mathur & VanderWeele, 2020). Instead of inferring bias from funnel plot symmetry, this approach quantifies the strength of publication bias required to attenuate the observed effect size to a null threshold. To account for dependence among effect sizes from the same article, this analysis used cluster-robust estimation with article specified as the clustering variable. As part of this analysis, we produced a significance funnel plot, which highlights the distribution of affirmative and nonaffirmative results relative to study precision. We further computed the *s*-value, defined as the minimum selection ratio necessary to shift the estimated effect size to zero. An *s*-value recorded as “not possible” indicates that the observed effect could not be explained away by publication bias.

## Results

### Study characteristics

Of the 994 records identified from databases and 428 records identified from other sources, 26 studies from 13 articles were included in the present meta-analysis. The total sample size across these studies was 542 participants. Study characteristics are summarized in Table 1.

### Overall paddle effect

**Fig. 3:**
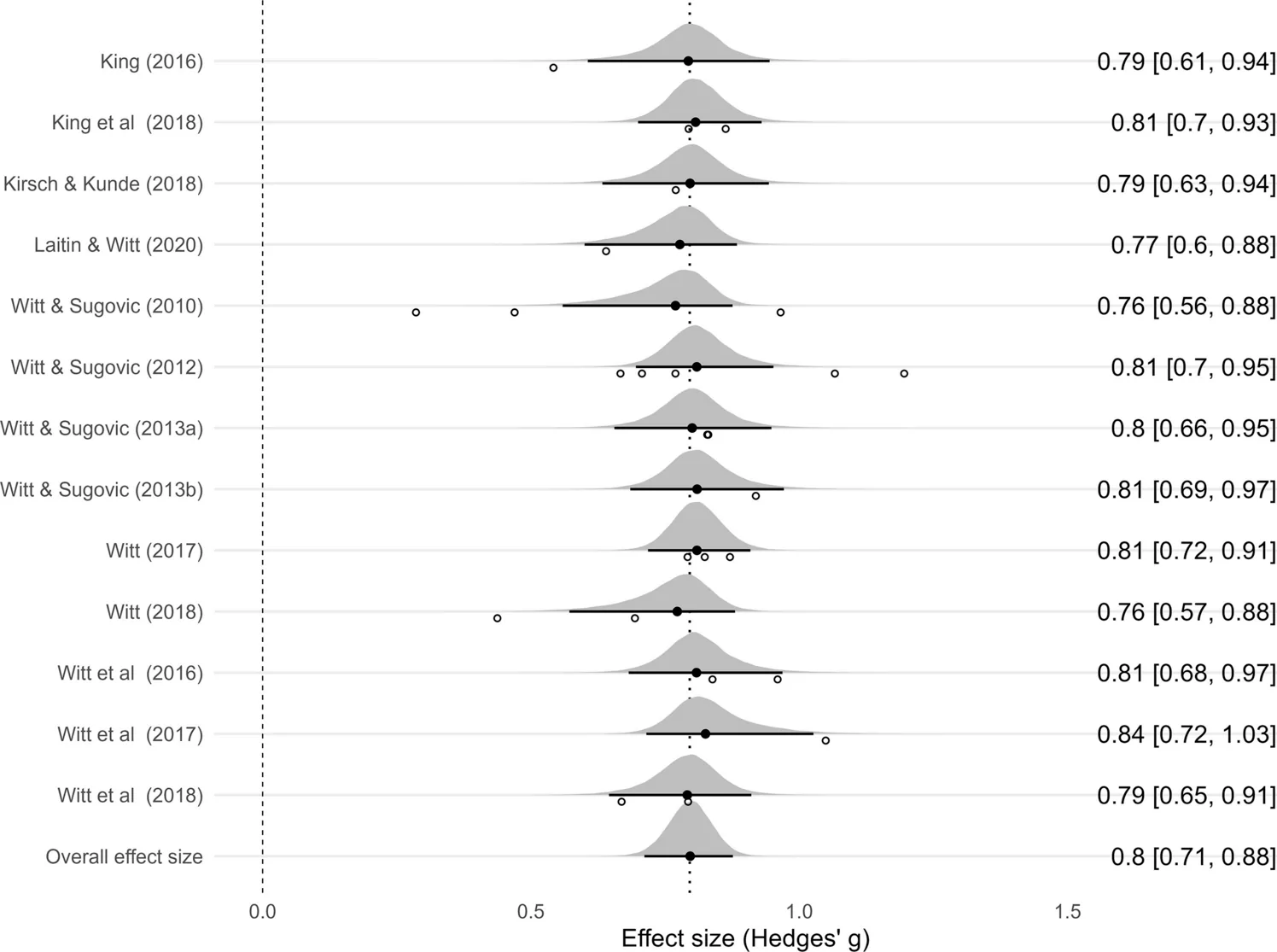
Forest plot of the paddle effect sizes. Black filled circles represent the estimated effect size for each article, and open circles represent the effect size for each study within articles. The gray areas indicate the posterior distributions, and the error bars represent the 95% CrIs

The Bayesian three-level meta-analysis showed an overall paddle effect of Hedges’ *g* = 0.80, 95% CrI [0.71, 0.88], with relatively low estimated heterogeneity at both the article level, τ_article_ = 0.06, 95% CrI [0.00, 0.16], and the study level, τ_study_ = 0.04, 95% CrI [0.00, 0.13]. BF_10_ = 1.02×10^9^, indicating extreme evidence for the existence of the overall paddle effect. That is, the data were 1.02×10^9^ times more likely under the alternative hypothesis of a non-zero effect than under the null hypothesis. The posterior effect size estimates for articles and studies are illustrated in the forest plot (Fig. 3).

For each model, the four MCMC chains showed good convergence, as all $\widehat{\mathrm{R}}$ values were equal to 1.00. Visual inspection of the trace plots also indicated that the chains were well-mixed.

### Sensitivity analysis

The leave-one-out sensitivity analysis revealed that the overall effect size ranged from 0.78 (95% CrI [0.69, 0.86]) to 0.82 (95% CrI [0.73, 0.90]) when removing one article (Level 3) at a time, and from 0.78 (95% CrI [0.69, 0.86]) to 0.81 (95% CrI [0.72, 0.89]) when removing one study (Level 2) at a time. This narrow range of variation indicates that the meta-analytic estimates were robust, as no single article or study exerted a high influence on the results.

### Moderator analysis

#### Paradigm

**Fig. 4:**
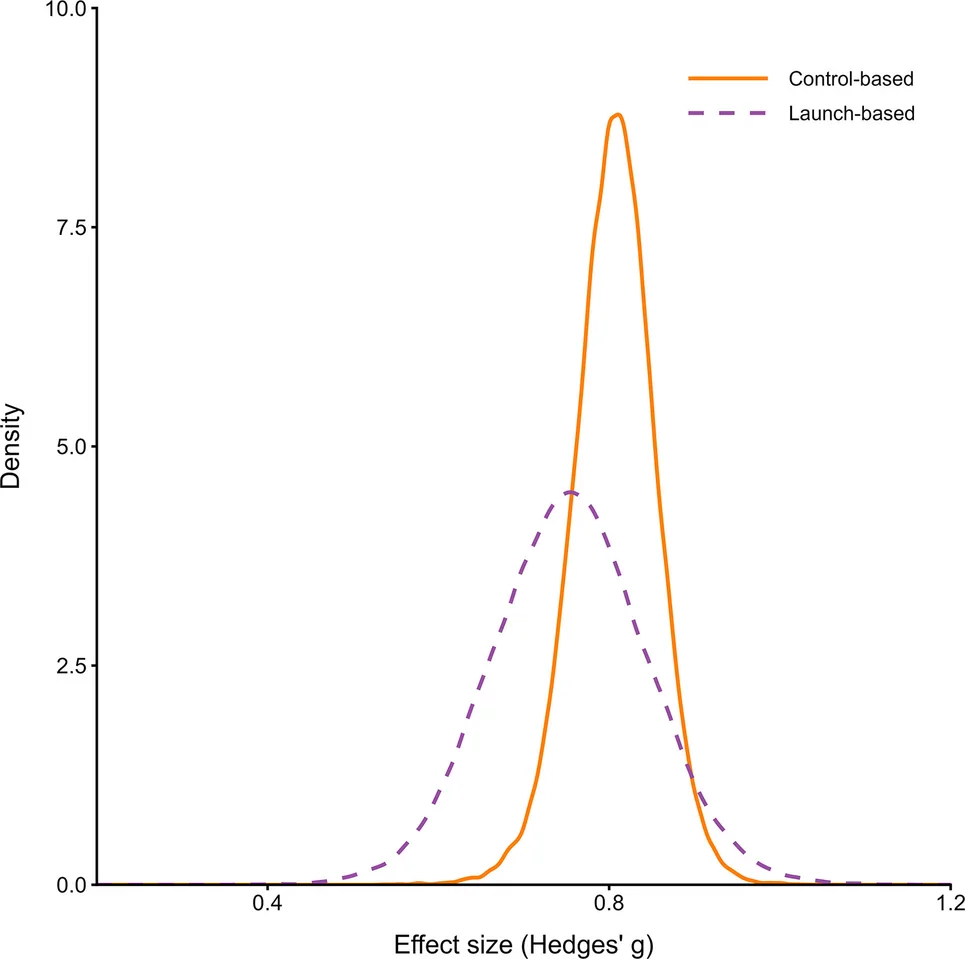
Posterior distributions of the effect sizes for the two paradigm levels under the Normal(0, 0.5) prior

The meta-regression analysis comparing the two levels of paradigm (control-based, *N*_outcome_ = 19 vs. launch-based, *N*_outcome_ = 7) provided moderate evidence against the presence of a moderating effect, β = −0.05, 95% CrI [−0.25, 0.15], BF_10_ = 0.23. The negative β indicates that the effect size for the launch-based paradigm was slightly smaller than that for the control-based paradigm. Across all prior specifications, the β estimates were stable and the BF_10_ values remained below 1. Under the narrower prior, β = −0.05, 95% CrI [−0.23, 0.14], BF_10_ = 0.43; under the wider prior, β = −0.05, 95% CrI [−0.25, 0.15], BF_10_ = 0.12. The posterior distributions of the effect sizes for the paradigm levels are displayed in Fig. 4.

#### Measure

**Fig. 5:**
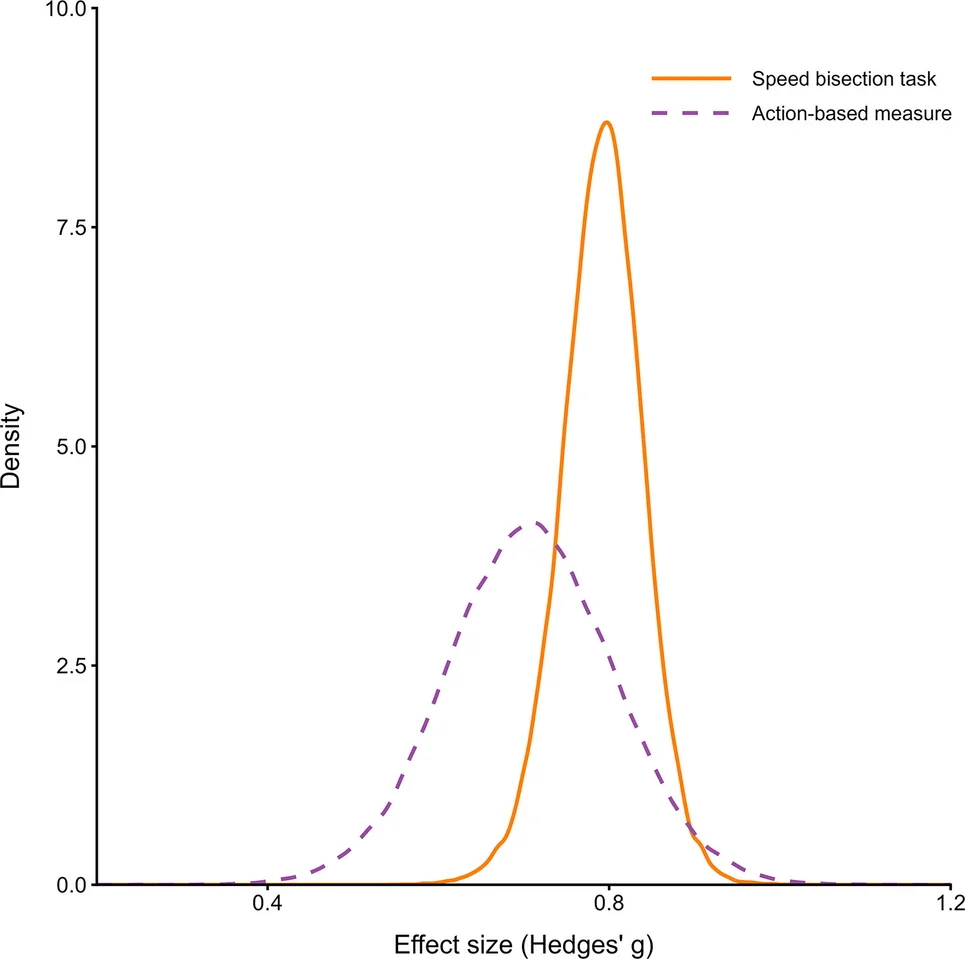
Posterior distributions of the effect sizes for the two measure levels under the Normal(0, 0.5) prior

Although the moderator of measure initially consisted of four categories, we removed speed rating task (*N*_outcome_ = 3) and speed comparison task (*N*_outcome_ = 2) due to the low number of outcomes, which would preclude reliable estimation of moderator effects. The final analysis retained two categories: speed bisection task (*N*_outcome_ = 20) and action-based measure (*N*_outcome_ = 6). The Bayesian meta-regression analysis provided moderate evidence against the presence of a moderating effect, β = −0.08, 95% CrI [−0.29, 0.12], BF_10_ = 0.30. The negative β indicates that the effect size for the action-based measure was slightly smaller than that for the speed bisection task. Across all prior specifications, the β estimates were stable and the BF_10_ values remained below 1. Under the narrower prior, β = −0.07, 95% CrI [−0.26, 0.12], BF_10_ = 0.53; under the wider prior, β = −0.09, 95% CrI [−0.29, 0.12], BF_10_ = 0.15. The posterior distributions of the effect sizes for the two measure levels are displayed in Fig. 5.

### Publication bias

**Fig. 6:**
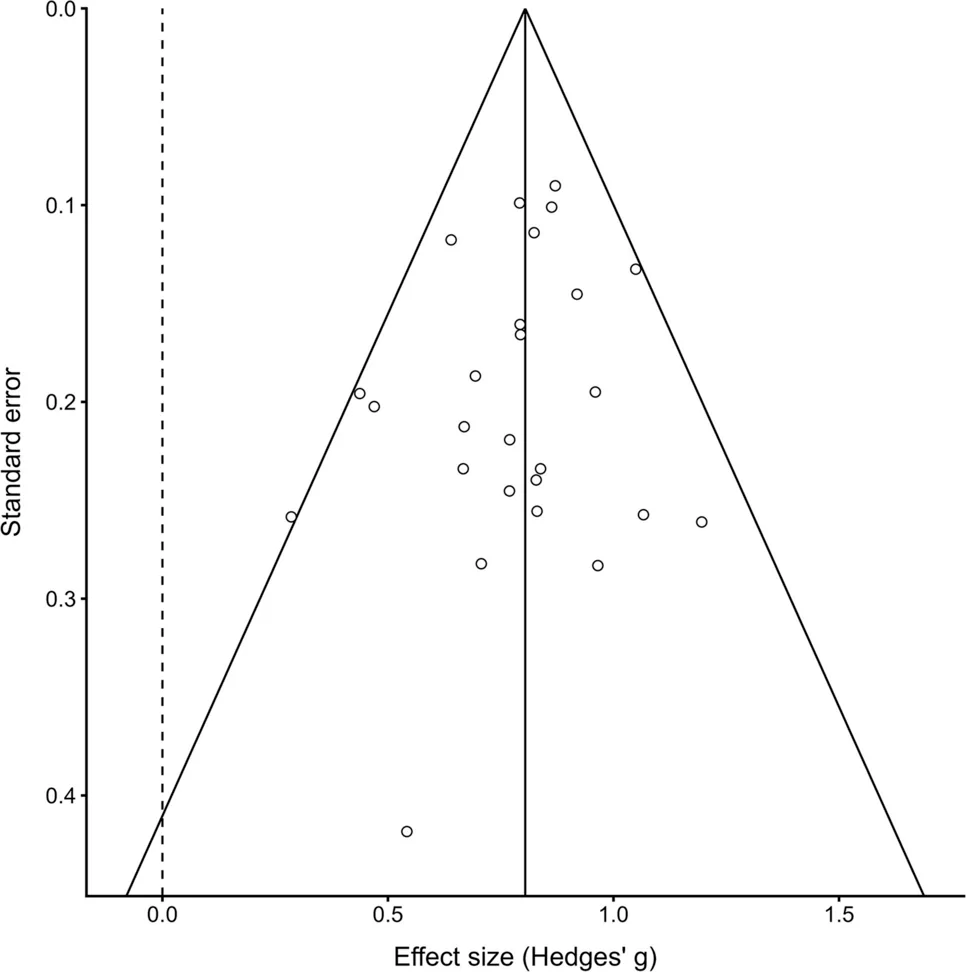
Funnel plot of the paddle effect sizes

**Fig. 7:**
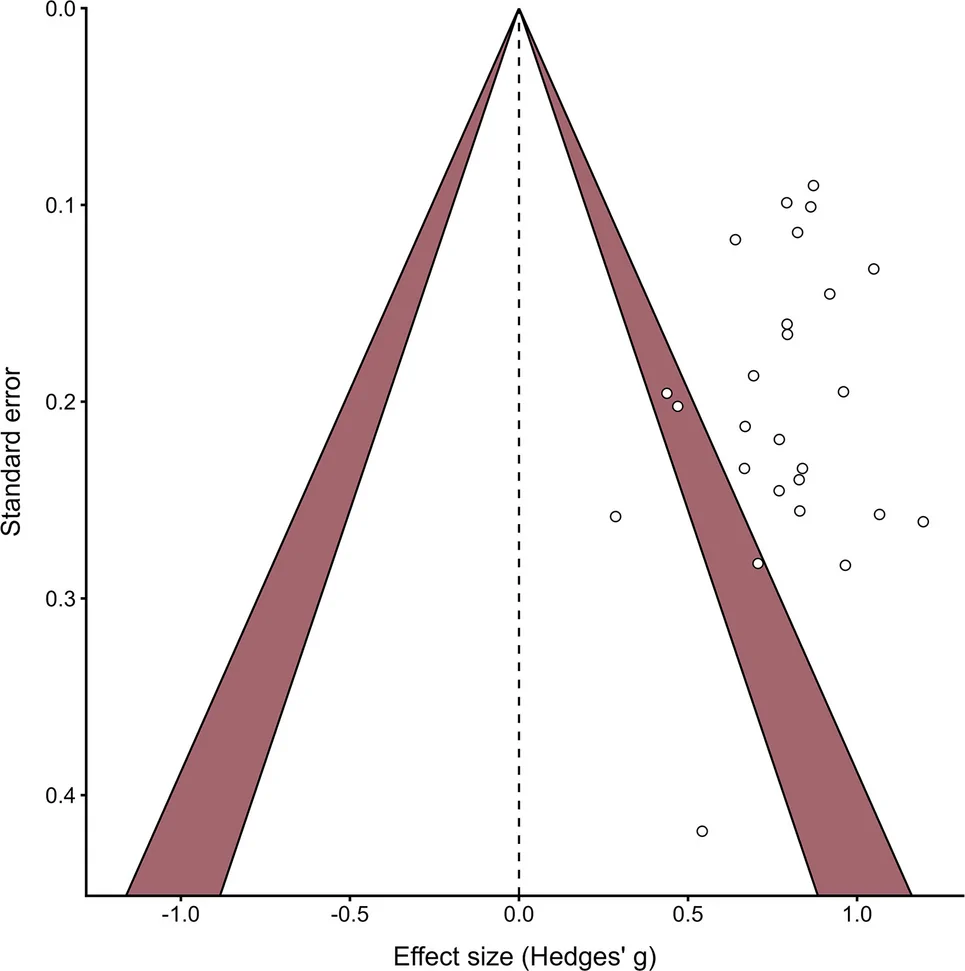
Contour-enhanced funnel plot of the paddle effect sizes. The central white region denotes *p* > .05; the red regions denote .01 ≤ *p* ≤ .05; and the outer white regions beyond the red contours denote *p* < .01. (Color figure online)

The funnel plot and contour-enhanced funnel plot are displayed in Figs. 6 and 7, respectively. The funnel plot was largely symmetrical, suggesting no strong evidence of publication bias based on visual inspection. The contour-enhanced funnel plot presents the same data but centered on zero and overlays significance contours, shown in red, indicating regions where results would be statistically significant. In our plot, most effect sizes were located within the right-hand significant regions rather than clustering around the significance thresholds. This pattern does not provide clear evidence for publication bias based on statistical significance. Consistently, the multilevel Egger’s regression test revealed no detectable asymmetry, *t*(24) = −0.89, *p* = .383.

**Fig. 8:**
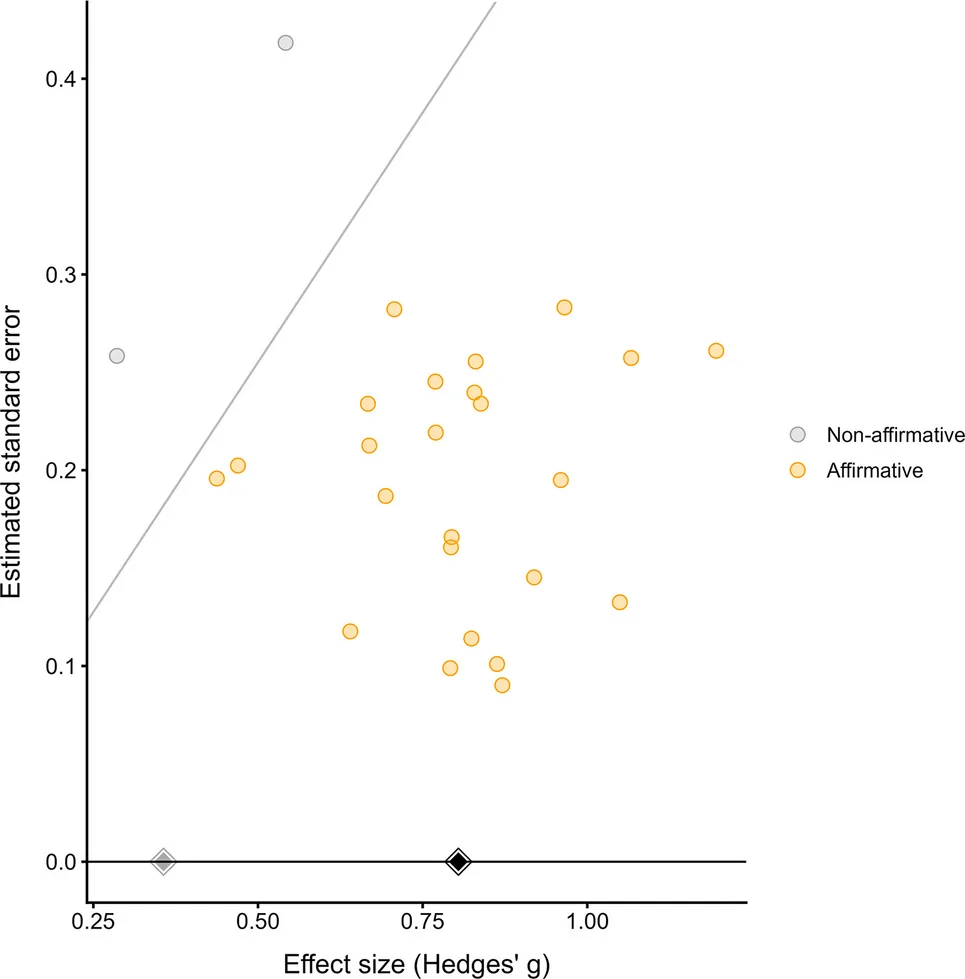
Significance funnel plot. Affirmative and nonaffirmative study-level effect sizes are color-coded relative to their standard errors. The two diamond markers indicate the independent point estimates within all studies and within the nonaffirmative studies, respectively

Using the sensitivity analysis approach, two study-level effect sizes were classified as nonaffirmative, while the remaining ones were affirmative (see Fig. 8). The *s*-value was reported as “Not possible,” indicating that, under the assumed selection model, the observed effect size could not be attenuated to zero by any plausible degree of publication bias. Furthermore, the selection ratio necessary to shift the 95% confidence interval to include zero was 24.55; that is, affirmative study-level results would have to be 24.55 times more likely to be selected for publication than nonaffirmative results. Together, these diagnostics provided converging evidence against a publication-bias account of the observed paddle effect.

## Discussion

The present meta-analysis provided extreme evidence supporting the existence of the paddle effect. The overall effect size was estimated to be Hedges’ *g* = 0.80, 95% CrI [0.71, 0.88], which conventionally reaches the threshold for a large effect (Cohen, 1988). This quantitative finding corroborates the notion of Tenhundfeld and Witt (2020), who characterized the paddle effect as a large effect based on empirical observations. However, it is still important to note that this result requires cautious interpretation, given that the credible interval includes values within the medium effect range.

Regarding heterogeneity, both between-article and between-study variability appeared to be low. Compared to the empirical distribution of heterogeneity in psychological meta-analyses (van Erp et al., 2017)—where τ typically ranges from 0 to 0.4—the heterogeneity observed here is consistent with relatively low variability. One plausible explanation is that most studies employed highly similar experimental methods, which likely constrained both between-article and between-study variability.

We further examined the potential influence of experimental paradigms on the paddle effect. If the effect were related to the degree of motor involvement, we would expect distinct effect sizes between the control-based paradigms (involving sustained manipulation) and launch-based paradigms (involving a single initiation). However, our Bayesian meta-regression yielded moderate evidence in favor of the null hypothesis, suggesting that the effect sizes across the two paradigms are comparable. This finding implies that the paddle effect may not be contingent upon the complexity of motor control. Perceivers scale spatial properties relative to the bodily effector matched to the anticipated action (Proffitt & Linkenauger, 2013). From this perspective, regardless of whether motor execution is continuous or discrete, the presence of a paddle may influence perceived target speed via its functional relevance to the task (Witt, 2021).

On the other hand, if the paddle effect were attributable to experimental demand characteristics (e.g., Durgin et al., 2009; Firestone, 2013), studies employing explicit speed bisection tasks should have yielded greater effect sizes than those using action-based measures. Bayesian moderate evidence does not support this hypothesis, suggesting that the paddle effect is unlikely to be explained by experimental demands.

Despite the ongoing debate regarding whether the paddle effect reflects a kind of coupling between action and perception, it is well established that these two systems interact reciprocally (Hommel et al., 2001). Functionally, frontoparietal circuits may serve as a critical interface between the perceptual and motor systems, thereby enabling coordinated perception-action processes (for a review, see Bosco et al., 2023). Indeed, the role of motor coding in perception (Hommel et al., 2001; Prinz, 1997) is evident even in simple hand movements (e.g., Buttaccio & Hahn, 2011). Such integration of signals from multiple modalities crucially contributes to optimized perception (Ernst & Banks, 2002; Ernst & Bülthoff, 2004). Taken together, this converging evidence suggests that if the paddle effect reflects an action-specific perceptual experience, the motor system is likely involved in the recalibration of speed perception to accommodate the functional demands of the task.

There are two primary limitations in the present study. First, a substantial number of the included studies originated from affiliated laboratories. We do not intend to imply publication bias, as our comprehensive diagnostics provide converging evidence against this concern. Rather, we emphasize the potential statistical challenges arising from the non-independence issue. In particular, these studies often employ overlapping experimental methods (Van den Noortgate et al., 2013), which may introduce data dependency and consequently limit the interpretation of our findings. Second, as with any meta-analysis, some eligible studies may not have been included due to the limited availability of sufficient statistics or unpublished findings. Nonetheless, the Bayesian framework helps address this challenge by enabling continuous analytical updates. In line with the principle that “today’s posterior is tomorrow’s prior” (Lindley, 1972, p. 2), this work serves as the foundation for a living meta-analysis. We encourage researchers to contribute missed or unpublished data via our public repository (https://osf.io/fxvbh/overview?view_only=a636518ac0f747eab10780f0e70f1e2d), which will support a continued characterization of the paddle effect.

Recently, research on action-specific perception has expanded into more athletic contexts, such as high jumping (Tanaka et al., 2018). Given that the present meta-analysis suggests a robust overall paddle effect, future work could examine whether this effect extends to more naturalistic ball-sport settings. Such work would help clarify the applied relevance of the paddle effect.

## Declarations

### Open practices statement

The data and R code are available online (https://osf.io/fxvbh/overview?view_only=a636518ac0f747eab10780f0e70f1e2d).
